# Osteoarthritis as a clinical marker of cardiovascular-kidney-metabolic multimorbidity: a population-based cohort study in China

**DOI:** 10.3389/fendo.2025.1660319

**Published:** 2025-09-09

**Authors:** Yue Zou, Yanyan Zhang, Xiujiang Sun, Wenbo Zhao, Guodong Zhang

**Affiliations:** ^1^ Department of Joint Surgery, Yantaishan Hospital, Yantai, Shandong, China; ^2^ East China Medical College of Fudan University, Shanghai, China

**Keywords:** osteoarthritis, CKM multimorbidity, multi-state mode, risk prediction, cohort study

## Abstract

**Background:**

Cardiovascular-kidney-metabolic (CKM) multimorbidity is increasingly recognized as a major global health concern. Although osteoarthritis (OA) is traditionally viewed as a degenerative joint condition, recent evidence suggests it may reflect systemic inflammation and metabolic dysfunction. However, its role as a potential clinical marker of CKM multimorbidity remains unclear.

**Methods:**

This population-based cohort study included 1,842 participants with osteoarthritis from a community registry in China. CKM multimorbidity was defined as the sequential development of cardiovascular disease (CVD), chronic kidney disease (CKD), and type 2 diabetes mellitus (T2DM). Participants were followed for incident CKM events. Logistic regression models estimated odds ratios (ORs) for new-onset, double, and triple CKM outcomes. A multi-state model was applied to assess progression trajectories between disease stages.

**Results:**

Over the study period, 32.6% of participants developed at least one CKM condition, 27.1% progressed to double CKM, and 5.4% developed triple CKM. Higher osteoarthritis burden was associated with increased risk of CKM multimorbidity. Adjusted ORs (95% CI) for new-onset, double, and triple CKM were 2.64 (2.33 – 3.00), 2.40 (2.11 – 2.72), and 1.49 (1.21 – 1.84), respectively. Multi-state modeling confirmed that osteoarthritis.

**Conclusion:**

Osteoarthritis is strongly associated with the onset and progression of CKM multimorbidity. As a clinically observable and common condition, OA may serve as an early indicator for identifying individuals at heightened risk of multi-organ metabolic decline. These findings support the integration of OA status into risk stratification frameworks for CKM disease prevention and management.

## 1 Introduction

Cardiovascular, kidney, and metabolic disorders frequently co-occur and share overlapping pathophysiological mechanisms, giving rise to the concept of cardiovascular-kidney-metabolic (CKM) multimorbidity (1, 2). As the global population ages and the burden of metabolic diseases continues to rise, the prevalence of CKM multimorbidity has grown significantly. Recent national data from the United States show that over 25% of adults are affected by at least one CKM-related condition, with nearly 10% experiencing overlapping diseases (3, 4). In China, the prevalence of type 2 diabetes mellitus (T2DM) (5), chronic kidney disease (CKD) (6), and cardiovascular disease (CVD) (7) continues to rise, particularly among older adults, where disease clustering is increasingly common. Identifying simple and accessible clinical indicators that can predict or reflect CKM multimorbidity would facilitate early detection and support integrated prevention strategies.

Traditionally viewed as a localized degenerative joint disease, osteoarthritis (OA) (8) is now increasingly recognized as a systemic condition associated with chronic low-grade inflammation (9), physical inactivity (10), obesity, and metabolic syndrome (11). In the past five years, numerous cohort and cross-sectional studies have demonstrated that OA shares similar metabolic pathways with CKM conditions. For instance, a Mendelian randomization study using UK Biobank data identified shared causal mechanisms between OA and cardiovascular diseases, involving cardiometabolic factors such as educational attainment, body mass index (BMI), smoking, and LDL cholesterol. Notably, BMI and smoking were found to mediate approximately one-third of the effect of education on OA risk (12). In 2023, a study utilizing data from the China Health and Retirement Longitudinal Study (CHARLS) reported that individuals with OA were more likely to develop chronic cardiometabolic conditions such as hypertension, diabetes, and CKD, suggesting that OA may be linked to CKM multimorbidity through shared risk mechanisms (13). However, despite growing evidence, few studies have investigated OA as a predictor of CKM disease progression, especially with regard to the accumulation of multimorbidity over time.

To address this gap, we conducted a population-based cohort study in China to examine the relationship between osteoarthritis and the incidence and progression of CKM multimorbidity. Using longitudinal follow-up data and a multi-state modeling framework, we evaluated whether OA is associated not only with the onset of individual CKM conditions—namely CVD, T2DM, and CKD—but also with transitions from a single disease to dual and triple multimorbidity. Our aim is to assess the potential utility of OA as a clinical marker for the early identification of individuals at elevated cardiometabolic risk.

## 2 Materials and methods

### 2.1 Study design and population

This population-based cohort study was conducted using data from adult patients diagnosed with osteoarthritis (OA) between January and December 2023 at community hospitals affiliated with Huashan Hospital, Fudan University, Shanghai, China. Eligible participants were aged ≥45 years and had a confirmed diagnosis of OA based on clinical symptoms and radiographic findings recorded in electronic health records.

Baseline demographic characteristics, laboratory results, and comorbidity status were extracted from standardized clinical records. Cardiovascular-kidney-metabolic (CKM) multimorbidity was defined as the coexistence or sequential development of cardiovascular disease (CVD), chronic kidney disease (CKD), and type 2 diabetes mellitus (T2DM), as identified by ICD-10 codes (e.g., I20–I25 for CVD, N18 for CKD, and E11 for T2DM).

Of the 8,621 patients initially screened, 4,381 were excluded due to missing baseline imaging or laboratory data or follow-up duration less than 6 months. Among the remaining 4,240 patients with complete information, 2,398 were excluded due to a prior diagnosis of any CKM condition at baseline. The final analytic cohort included 1,842 OA patients who were free of CVD, CKD, and T2DM at baseline and were followed longitudinally for incident CKM multimorbidity.

### 2.2 Definition of osteoarthritis and CKM multimorbidity

Osteoarthritis (OA) was defined based on clinical diagnosis by a licensed physician, confirmed with radiographic findings indicative of degenerative joint disease, including joint space narrowing, osteophyte formation, and subchondral sclerosis. OA severity was stratified into quartiles based on a composite symptom score derived from patient-reported joint pain frequency, stiffness duration, and physical function impairment. All included diagnoses conformed to the American College of Rheumatology criteria. Only patients newly diagnosed between January and December 2023 were eligible. CKM multimorbidity was defined as the sequential development of cardiovascular disease (CVD), chronic kidney disease (CKD), and type 2 diabetes mellitus (T2DM) during the follow-up period. The first CKM event was defined as the initial diagnosis of any one of these three conditions. Double CKM was defined as the coexistence of any two, and triple CKM was defined as the presence of all three. CVD included ischemic heart disease, myocardial infarction, or stroke, as identified by ICD - 10 codes I20–I25 and I60–I69. CKD was identified based on an estimated glomerular filtration rate (eGFR) below 60 mL/min/1.73 m² or a physician-confirmed diagnosis (ICD - 10 N18). T2DM was defined by physician diagnosis, fasting glucose ≥7.0 mmol/L, HbA1c ≥6.5%, or the use of glucose-lowering medication (ICD - 10 E11).

Participants were followed from the date of OA diagnosis until the first occurrence of any CKM condition, death, loss to follow-up, or the end of the observation period (December 2024). The trajectory of disease accumulation was evaluated across four stages: CKM-free status at baseline, onset of a first CKM event, development of double CKM, and progression to triple CKM. Potential mechanistic pathways linking OA to CKM multimorbidity include systemic inflammation, physical inactivity, and obesity-related metabolic dysfunction, which may accelerate vascular, renal, and glycemic deterioration over time.

### 2.3 Covariates

Baseline covariates were selected based on prior literature and clinical relevance to cardiometabolic outcomes. Demographic variables included age, sex, and educational attainment. Behavioral factors included smoking status (never, former, current) and physical activity status, defined as engagement in regular moderate or vigorous activity at least three times per week. Clinical measurements included systolic and diastolic blood pressure, body mass index (BMI), and laboratory markers such as low-density lipoprotein (LDL) cholesterol, high-density lipoprotein (HDL) cholesterol, and triglycerides. Inflammatory markers included high-sensitivity C-reactive protein (CRP) and erythrocyte sedimentation rate (ESR), which were measured using standardized assays at baseline. History of hypertension was defined by physician diagnosis or the use of antihypertensive medications. All laboratory tests and clinical assessments were conducted at the time of OA diagnosis and recorded in the hospital’s electronic medical record system using standardized protocols. These covariates were included as potential confounders in the regression and multi-state models to adjust for baseline differences and isolate the association between osteoarthritis and CKM disease progression.

Although comprehensive clinical and laboratory data were available, certain potential confounders such as detailed dietary intake, statin or antidiabetic medication use, and individual socioeconomic status were not systematically captured in the electronic health record system. Therefore, these variables could not be included in the primary multivariable models.

### 2.4 Statistical analysis

Baseline characteristics were summarized across quartiles of osteoarthritis severity using means with standard deviations for continuous variables and counts with percentages for categorical variables. Group differences were assessed using analysis of variance for normally distributed variables, the Kruskal–Wallis test for skewed variables, and chi-square tests for categorical variables. Multivariable logistic regression models were used to examine the association between osteoarthritis severity and the risk of new-onset, double, and triple CKM multimorbidity, with results expressed as odds ratios (ORs) and 95% confidence intervals (CIs). Both crude and adjusted models were constructed, with adjustment for age, sex, BMI, blood pressure, lipid profiles, inflammatory markers, smoking, physical activity, and hypertension history.

A four-state progressive multi-state model was constructed to capture the sequential accumulation of cardiovascular-kidney-metabolic (CKM) conditions. The model comprised the following states: (1) CKM-free at baseline, (2) first CKM event (defined as initial diagnosis of CVD, CKD, or T2DM), (3) double CKM (coexistence of any two conditions), and (4) triple CKM (presence of all three). Transitions were restricted to a forward-only structure with no backward or skip transitions allowed. This progressive design reflects the clinical trajectory of disease burden accumulation over time. The state structure and allowable transitions are illustrated in **Supplementary Figure S1**.

To further evaluate the longitudinal progression of CKM multimorbidity, a multi-state model was constructed to estimate the hazard of transition from a CKM-free state to first CKM event, from first to double CKM, and from double to triple CKM. Additional models examined transition probabilities based on the initial CKM subtype (CVD, CKD, or T2DM). The transition matrix allowed only forward movements: from CKM-free (State 0) to first CKM (States 1a–1c), then to corresponding double CKM states (States 2a–2c), and finally to the triple CKM state (State 3). Non-sequential transitions—such as from CKM-free to double or triple CKM, or from double CKM to earlier states—were not permitted and set to zero in the transition matrix. This clinically progressive structure was specified using the “trans” function in the mstate package. A Markov assumption was adopted, where transition intensities depend only on the current state and not on the time already spent in that state. This aligns with the default clock-forward approach in the mstate package. We did not apply a semi-Markov or clock-reset model, as sojourn time effects were not the primary focus of this analysis.

All covariates included in the multi-state models were time-fixed and derived from baseline assessments. Time-updated covariates were not incorporated due to the lack of repeated measurements across follow-up intervals. As the primary aim was to assess the prognostic relevance of baseline osteoarthritis severity and systemic risk factors, baseline values were used consistently to minimize modeling complexity and ensure interpretability. We acknowledge that time-varying covariates may provide additional insights in longer follow-up settings.

The multi-state model was fitted using the “mstate” package in R (version 4.4.1), and transition dates were aligned using a 0.5-day offset strategy to account for same-day state changes, as previously recommended. Dose–response relationships were evaluated using restricted cubic spline analysis. Sensitivity analyses were performed by varying the time intervals between disease state transitions and by expanding the definition of CKM to include obesity, dyslipidemia, heart failure, and atrial fibrillation. All statistical tests were two-sided, with a significance threshold set at P < 0.05.

Participants with incomplete baseline data or insufficient follow-up duration (<6 months) were excluded from the analysis, resulting in a complete-case cohort. Censoring was handled by right-censoring individuals at the time of loss to follow-up, death, or end of the observation period. Death was not modeled as a competing risk or absorbing state due to lack of systematic mortality recording in the database. As such, transitions were modeled conditional on survival, and findings are interpreted within that context.

### 2.5 Multi-state model analysis

We constructed a progressive multi-state model to characterize the temporal accumulation of CKM multimorbidity among osteoarthritis (OA) patients. The model defined four sequential health states: (1) CKM-free; (2) single CKM condition (CVD, CKD, or T2DM); (3) double CKM; and (4) triple CKM. Direct transitions between non-adjacent states were not permitted. Transition times were calculated as the intervals from OA diagnosis to the occurrence of each CKM component. For participants who developed multiple conditions on the same date, a 0.5-day offset was introduced to preserve chronological order, a method consistent with prior epidemiological modeling practices. All transition-specific Cox models converged successfully, with no signs of multicollinearity or boundary issues. Competing risk from mortality was not modeled due to limited availability of death records; however, a sensitivity analysis excluding patients with early loss to follow-up (<3 months) showed similar trends.

## 3 Results

### 3.1 Baseline characteristics of the study population

A total of 1,842 participants with clinically diagnosed osteoarthritis were included in the final analysis. The mean age was 64.95 years (SD 8.16), and 56.2% were female. Participants were stratified into quartiles according to osteoarthritis severity, with 461 individuals in each quartile. As shown in **Table 1**, participants in higher quartiles had significantly higher body mass index (BMI), increasing from 21.93 kg/m² in Q1 to 28.18 kg/m² in Q4 (P < 0.001). Systolic and diastolic blood pressure also showed a positive trend across quartiles (P < 0.001 for both). However, there were no significant differences in LDL cholesterol, HDL cholesterol, or triglyceride levels.

**Table 1:** Baseline characteristics of participants according to quartiles of osteoarthritis severity (N=1,842).

Characteristic	Overall (N=1842)	Q1	Q2	Q3	Q4	P value
Demographics						
BMI, kg/m^2^	25.01 (2.46)	21.93 (1.16)	24.19 (0.48)	25.76 (0.49)	28.18 (1.26)	<0.001
Age, years	64.95 (8.16)	65.39 (8.63)	64.73 (8.05)	64.91 (7.61)	64.76 (8.31)	0.5887
Sex: Male	807 (43.81)	205 (44.47)	211 (45.87)	208 (45.22)	183 (39.70)	
Sex: Female	1035 (56.19)	256 (55.53)	249 (54.13)	252 (54.78)	278 (60.30)	0.2202
Clinical Measurements						
Systolic blood pressure, mmHg	120.23 (10.19)	117.43 (9.59)	119.76 (10.46)	120.63 (9.67)	123.11 (10.24)	<0.001
Diastolic blood pressure, mmHg	74.86 (5.86)	73.94 (5.77)	74.59 (5.84)	75.18 (5.83)	75.71 (5.86)	<0.001
LDL cholesterol, mmol/L	2.79 (0.60)	2.82 (0.60)	2.77 (0.59)	2.79 (0.59)	2.78 (0.63)	0.4861
HDL cholesterol, mmol/L	1.20 (0.29)	1.20 (0.28)	1.20 (0.28)	1.22 (0.28)	1.18 (0.31)	0.2774
Triglycerides, mmol/L	1.41 (0.49)	1.42 (0.51)	1.38 (0.49)	1.42 (0.50)	1.40 (0.48)	0.6127
Inflammatory Markers						
C-reactive protein, mg/L	1.00 (0.99)	1.00 (1.07)	0.95 (0.92)	1.07 (1.03)	0.99 (0.93)	0.2755
Erythrocyte sedimentation rate, mm/h	20.46 (9.37)	20.38 (9.46)	20.88 (9.07)	20.88 (9.32)	19.70 (9.60)	0.1823
Behavioral Factors						
Physical activity (yes)	1200 (65.15)	330 (71.58)	289 (62.83)	283 (61.52)	298 (64.64)	0.0067
Disease History						
Osteoarthritis (yes)	1842 (100.00)	461 (100.00)	460 (100.00)	460 (100.00)	461 (100.00)	1
Chronic kidney disease (yes)	196 (10.64)	53 (11.50)	39 (8.48)	55 (11.96)	49 (10.63)	0.3266
Cardiovascular disease (yes)	198 (10.75)	51 (11.06)	51 (11.09)	51 (11.09)	45 (9.76)	0.8906
Type 2 diabetes (yes)	206 (11.18)	53 (11.50)	51 (11.09)	52 (11.30)	50 (10.85)	0.9907
CKM Outcomes						
New-onset CKM (any)	600 (32.57)	60 (13.02)	90 (19.57)	150 (32.61)	300 (65.08)	<0.001
Double CKM	500 (27.14)	51 (11.06)	75 (16.30)	125 (27.17)	249 (54.01)	<0.001
Triple CKM	100 (5.43)	13 (2.82)	14 (3.04)	32 (6.96)	41 (8.89)	<0.001

Values are shown as mean (SD) for continuous variables and n (%) for categorical variables. OA severity was assessed at baseline using a composite symptom score (joint pain, stiffness, and function impairment), with quartiles (Q1–Q4) indicating increasing severity. P values were calculated using ANOVA or chi-square tests, as appropriate.

BMI, body mass index; SBP, systolic blood pressure; DBP, diastolic blood pressure; LDL, low-density lipoprotein; HDL, high-density lipoprotein; CRP, C-reactive protein; ESR, erythrocyte sedimentation rate; CKM, cardiovascular-kidney-metabolic; CVD, cardiovascular disease; CKD, chronic kidney disease; T2DM, type 2 diabetes mellitus.

**Figure 1:**
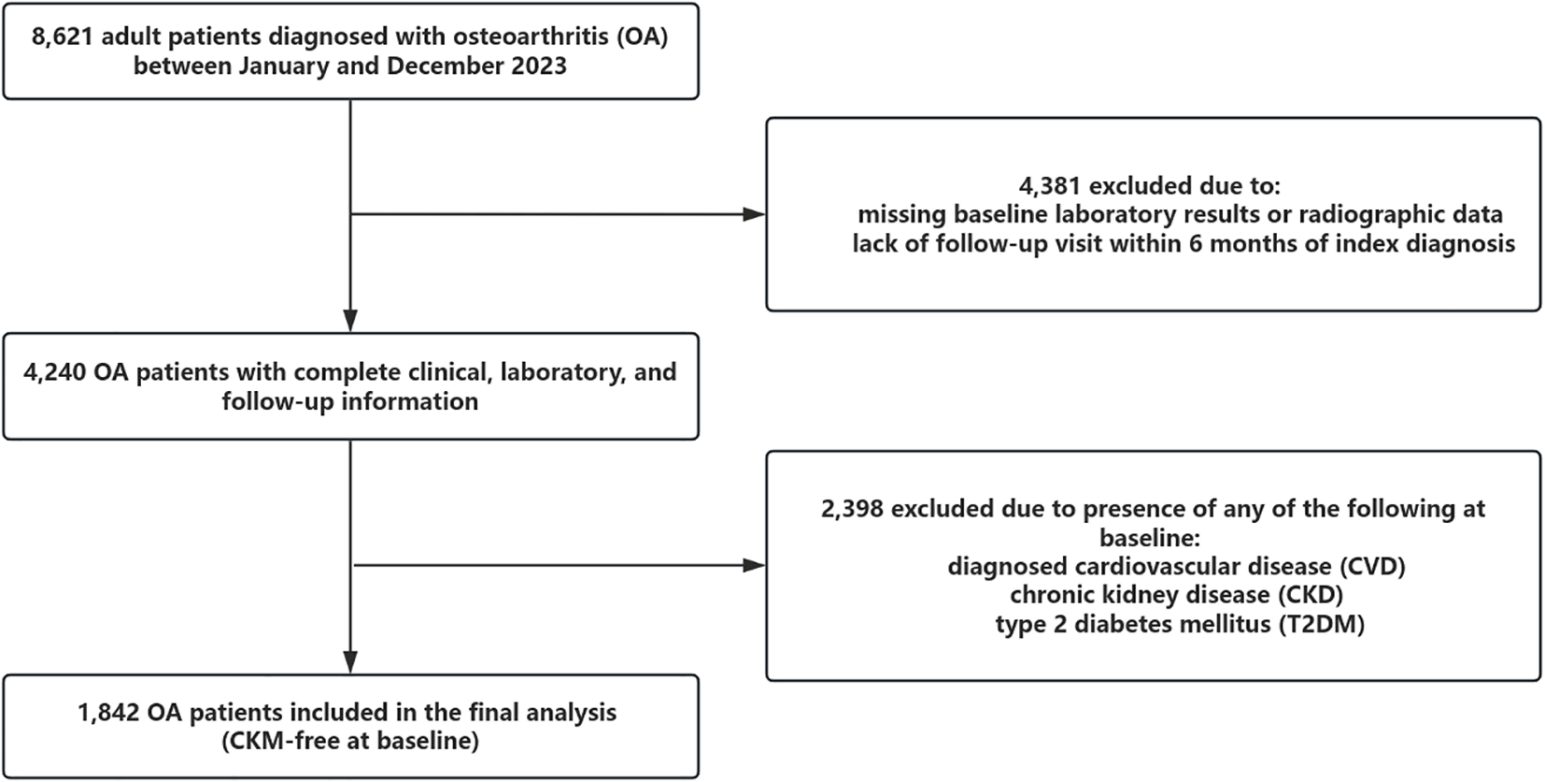
This figure shows the study flowchart of participant selection. A total of 8,621 patients diagnosed with osteoarthritis (OA) were initially screened. After excluding 4,381 individuals due to missing baseline data or insufficient follow-up, 4,240 patients remained. Among them, 2,398 were excluded due to pre-existing cardiovascular disease (CVD), chronic kidney disease (CKD), or type 2 diabetes mellitus (T2DM) at baseline. The final analytic cohort included 1,842 participants who were free of CKM conditions at baseline and were followed prospectively for incident CKM outcomes.

The prevalence of physical activity decreased with increasing OA severity, from 71.6% in Q1 to 64.6% in Q4 (P = 0.007). Inflammatory markers, including C-reactive protein and erythrocyte sedimentation rate, did not differ significantly across quartiles. Baseline prevalence of individual CKM diseases—CVD, CKD, and T2DM—was consistent across groups, ranging from 8.5% to 12.0%. However, the cumulative burden of CKM conditions during follow-up exhibited a strong dose-response relationship with OA severity. New-onset CKM occurred in 32.6% of the cohort, with rates rising from 13.0% in Q1 to 65.1% in Q4 (P < 0.001). Double CKM occurred in 27.1%, and triple CKM in 5.4%, both showing significant upward trends (P < 0.001). The study flow diagram in **Figure 1** illustrates the selection process and the progression of CKM outcomes across quartiles.

### 3.2 Association between osteoarthritis and CKM multimorbidity

**Figure 2:**
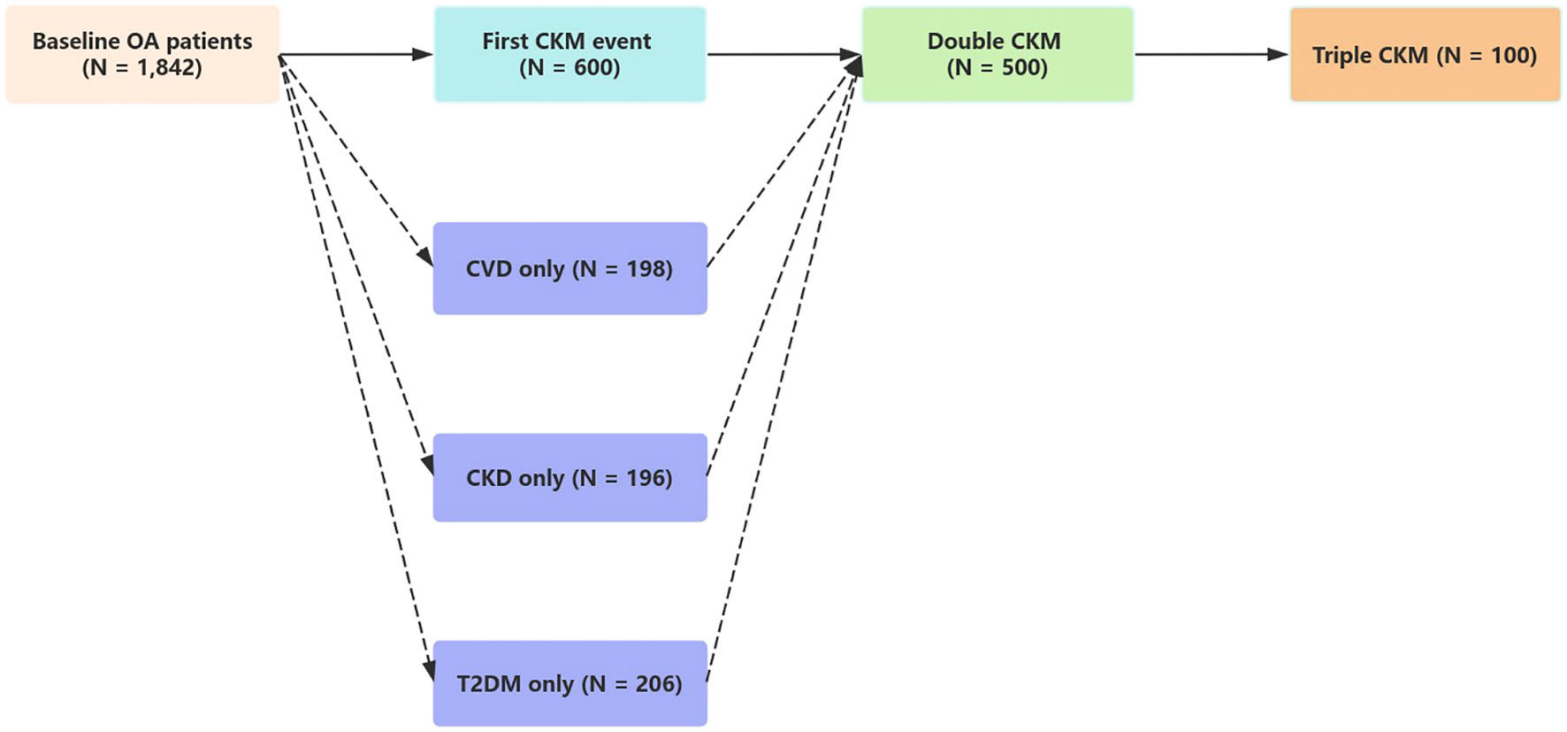
This figure displays the prevalence of CKM multimorbidity across quartiles of osteoarthritis (OA) severity among 1,842 participants. The prevalence of new-onset CKM increased from 13.02% in the lowest quartile (Q1) to 65.08% in the highest quartile (Q4). Similarly, the prevalence of double CKM rose from 11.06% in Q1 to 54.01% in Q4, and the prevalence of triple CKM increased from 2.82% in Q1 to 8.89% in Q4. All trends across quartiles were statistically significant (P for trend < 0.001), indicating a strong dose–response relationship between OA severity and CKM burden.

**Figure 3:**
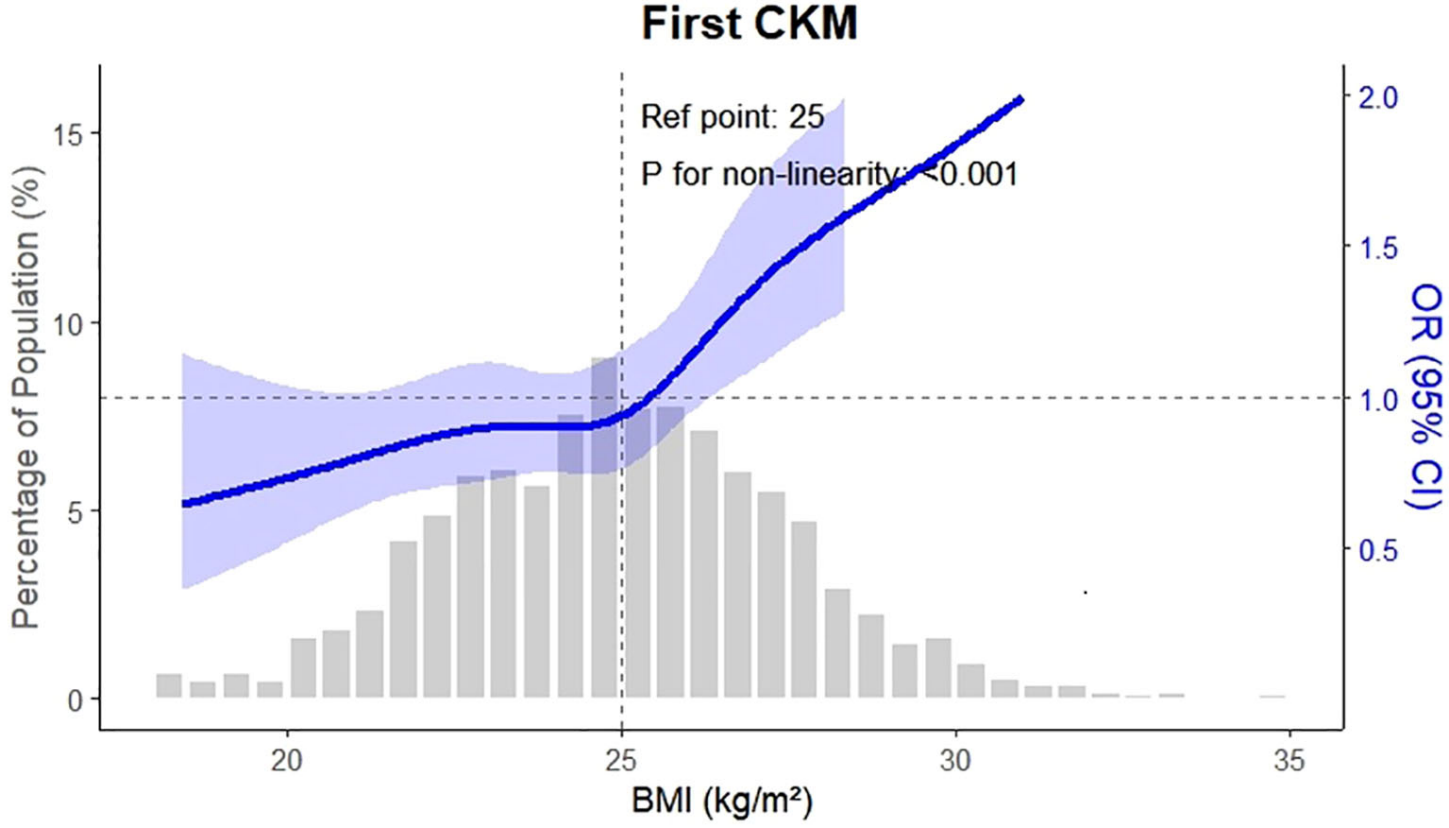
This figure presents the adjusted odds ratios (ORs) for CKM multimorbidity outcomes by osteoarthritis (OA) severity. Compared with participants in the lowest OA quartile (Q1), those in higher quartiles had significantly increased odds of developing CKM outcomes. The adjusted OR for new-onset CKM was 2.64 (95% confidence interval \[CI]: 2.33–3.00), for double CKM was 2.40 (95% CI: 2.11–2.72), and for triple CKM was 1.49 (95% CI: 1.21–1.84). These estimates were derived from multivariable logistic regression models adjusted for age, sex, body mass index, blood pressure, lipid levels, inflammatory markers, physical activity, and hypertension history.

**Figure 2** illustrates the prevalence of CKM conditions across OA severity quartiles, complementing **Figure 3**, which presents the adjusted odds ratios to quantify the magnitude of these associations. The association between osteoarthritis severity and CKM multimorbidity was examined using logistic regression models. As shown in **Table 2**, increasing OA severity was significantly associated with a higher risk of developing new-onset CKM, as well as progressing to double or triple CKM. In the adjusted model, the odds of new-onset CKM were more than two-and-a-half times higher among individuals in higher OA quartiles compared to those in the lowest quartile (OR: 2.64, 95% CI: 2.33 – 3.00; P < 0.001). Similarly, the odds of double and triple CKM were significantly elevated (OR: 2.40, 95% CI: 2.11 – 2.72; OR: 1.49, 95% CI: 1.21 – 1.84; both P < 0.001).

**Table 2:** Association between osteoarthritis severity and CKM multimorbidity outcomes: crude and adjusted logistic regression models.

Outcome	Crude OR (95% CI)	Crude P	Crude Pseudo R²	Adjusted OR (95% CI)	Adjusted P	Adjusted Pseudo R²
New-onset CKM (any)	2.59 (2.29–2.93)	<0.001	0.125	2.64 (2.33–3.00)	<0.001	0.13
Double CKM	2.33 (2.06–2.63)	<0.001	0.102	2.40 (2.11–2.72)	<0.001	0.108
Triple CKM	1.46 (1.19–1.78)	<0.001	0.017	1.49 (1.21–1.84)	<0.001	0.028
Cardiovascular disease	0.95 (0.82–1.10)	0.4724	0	0.96 (0.82–1.12)	0.6175	0.005
Chronic kidney disease	1.00 (0.87–1.16)	0.9593	0	1.00 (0.86–1.17)	0.9796	0.006
Type 2 diabetes	0.95 (0.82–1.09)	0.4502	0	0.93 (0.80–1.08)	0.3536	0.015

Odds ratios (ORs) and 95% confidence intervals (CIs) were estimated using logistic regression. Adjusted models were controlled for age, sex, body mass index (BMI), systolic and diastolic blood pressure, LDL and HDL cholesterol, triglycerides, C-reactive protein, erythrocyte sedimentation rate, physical activity, smoking status, and history of hypertension. New-onset CKM refers to the first occurrence of any of the following: cardiovascular disease, chronic kidney disease, or type 2 diabetes mellitus. Double CKM refers to the coexistence of any two of the three conditions, and triple CKM refers to the presence of all three.

Pseudo R² represents model goodness-of-fit.

CKM, cardiovascular-kidney-metabolic; OR, odds ratio; CI, confidence interval.

In contrast, osteoarthritis was not significantly associated with the development of individual CKM components when evaluated separately. The adjusted odds ratios were 0.96 (95% CI: 0.82 – 1.12) for cardiovascular disease, 1.00 (95% CI: 0.86 – 1.17) for chronic kidney disease, and 0.93 (95% CI: 0.80 – 1.08) for type 2 diabetes mellitus, with all P values > 0.05. These null results suggest that OA may not strongly influence any single CKM condition in isolation. However, the consistent association with CKM clustering indicates that OA could promote multimorbidity through synergistic or cumulative systemic pathways, possibly involving chronic inflammation or metabolic dysregulation.

### 3.3 Progression of CKM multimorbidity: multi-state model analysis

**Figure 4:**
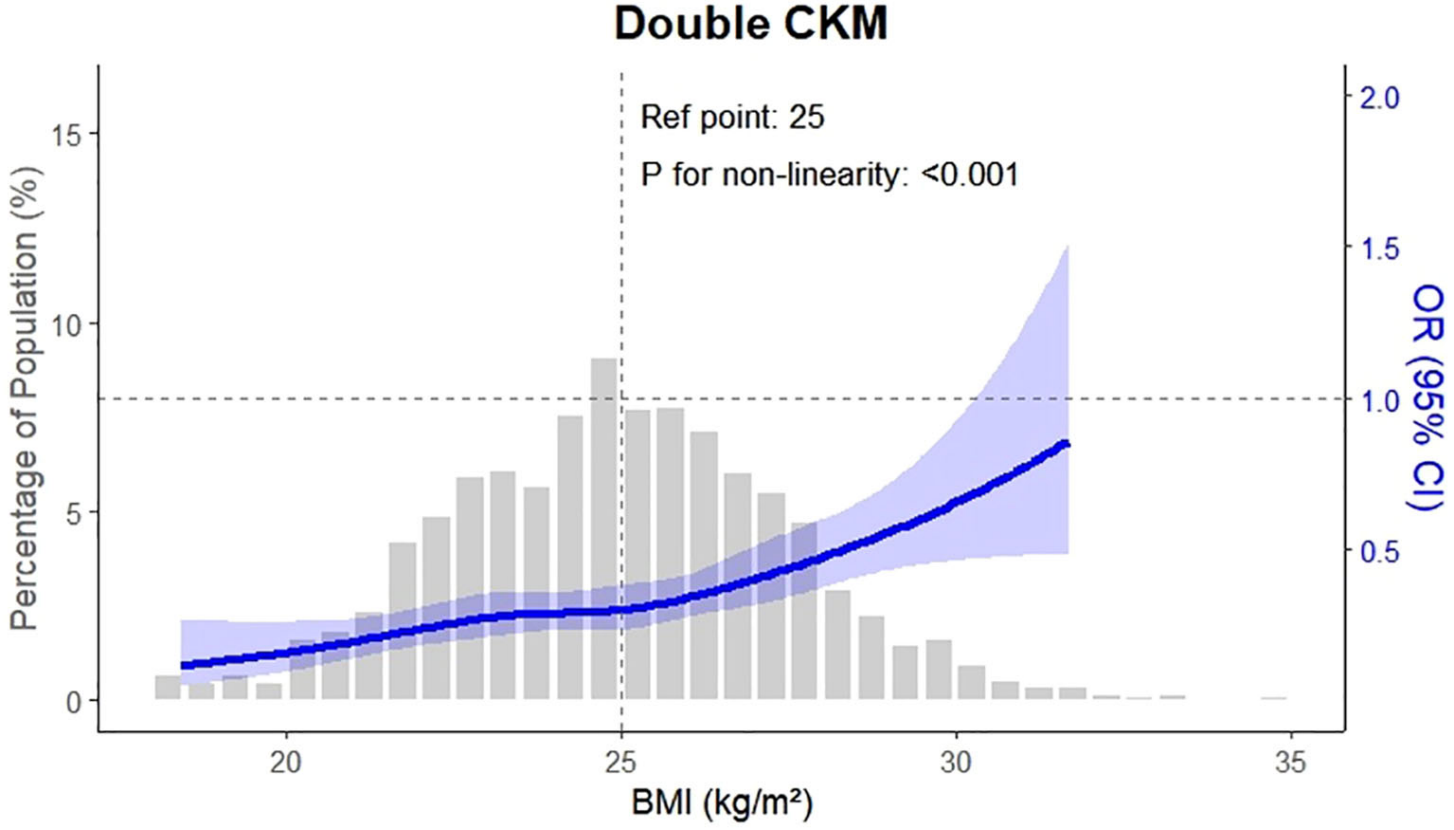
This figure illustrates the progression of CKM multimorbidity in patients with osteoarthritis using a multi-state model. Among 1,842 participants free of CKM conditions at baseline, 600 developed a first CKM condition during follow-up. Of these, 500 progressed to double CKM, and 100 further progressed to triple CKM. The adjusted hazard ratio (HR) for the transition from baseline to first CKM condition was 2.64 (95% CI: 2.33–3.00), from first to double CKM was 2.40 (95% CI: 2.11–2.72), and from double to triple CKM was 1.49 (95% CI: 1.21–1.84). These estimates were based on time-to-event analyses using the multi-state modeling framework.

**Figure 5:**
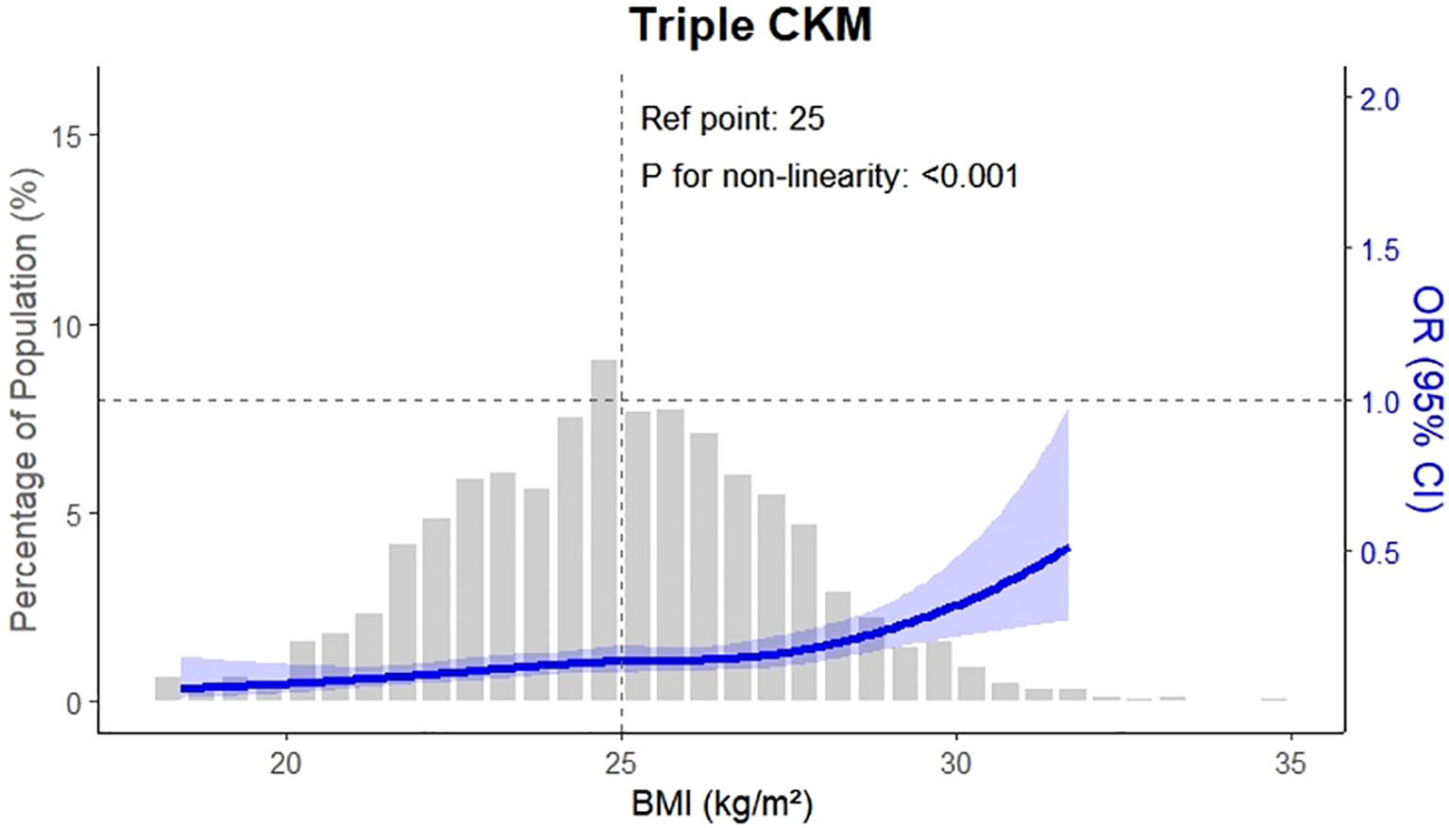
This figure shows the hazard ratios (HRs) for progression to double CKM multimorbidity according to the type of the first CKM event. Among participants who developed a first CKM condition, those whose first event was type 2 diabetes mellitus (T2DM) had an HR of 1.69 (95% CI: 1.21–2.36) for progressing to double CKM. For those whose first event was cardiovascular disease (CVD), the HR was 2.31 (95% CI: 1.56–3.43), and for those with chronic kidney disease (CKD) as the first event, the HR was 2.86 (95% CI: 1.85–4.41). All models were adjusted for demographic, behavioral, and clinical covariates using a multi-state modeling approach.

**Figures 4** and **5** both depict state transitions in CKM progression, but **Figure 4** presents the overall transition dynamics, whereas **Figure 5** stratifies transitions by the type of initial CKM event. To evaluate longitudinal transitions in CKM disease burden, we applied a multi-state model to track progression from a CKM-free baseline to new-onset, double, and triple CKM stages. As illustrated in **Figure 4**, among 1,842 osteoarthritis patients without baseline CKM disease, 600 (32.6%) developed a first CKM condition during follow-up. Of these, 500 individuals progressed to double CKM, and 100 subsequently developed triple CKM.

As shown in **Table 3**, osteoarthritis severity was significantly associated with each transition along the CKM multimorbidity trajectory. The hazard ratio (HR) for the transition from baseline to first CKM disease was 2.64 (95% CI: 2.33 – 3.00, P < 0.001). Among those who developed a first CKM condition, the HR for progression to double CKM was 2.40 (95% CI: 2.11 – 2.72, P < 0.001). Additionally, the HR for progression from double to triple CKM was 1.49 (95% CI: 1.21 – 1.84, P < 0.001), indicating a sustained influence of osteoarthritis across successive disease states.

**Table 3:** Hazard ratios for transitions in CKM multimorbidity among participants with osteoarthritis based on multi-state model.

Transition	HR (95% CI)	P value
Model 1: Baseline → First CKM	2.64 (2.33–3.00)	<0.001
Model 1: First CKM → Double CKM	2.40 (2.11–2.72)	<0.001
Model 1: Double CKM → Triple CKM	1.49 (1.21–1.84)	<0.001
Model 2: Baseline → CVD	0.96 (0.82–1.12)	0.6175
Model 2: Baseline → T2DM	0.93 (0.80–1.08)	0.3536
Model 2: Baseline → CKD	1.00 (0.86–1.17)	0.9796
Model 2: T2DM → Double CKM	1.69 (1.21–2.36)	0.0022
Model 2: CVD → Double CKM	2.31 (1.56–3.43)	<0.001
Model 2: CKD → Double CKM	2.86 (1.85–4.41)	<0.001
Model 2: Double CKM → Triple CKM	0.76 (0.59–0.97)	0.0302

Hazard ratios (HRs) and 95% confidence intervals (CIs) were derived from multi-state models evaluating transitions between CKM disease stages. Model 1 assessed transitions from baseline to first CKM condition, then to double and triple CKM. Model 2 evaluated transitions from baseline to individual CKM components (CVD, T2DM, or CKD) and subsequent progression to double CKM.

CKM, cardiovascular-kidney-metabolic; CVD, cardiovascular disease; CKD, chronic kidney disease; T2DM, type 2 diabetes mellitus; HR, hazard ratio; CI, confidence interval.

Notably, when comparing crude and adjusted models for the transition from double to triple CKM, a discrepancy in hazard ratios was observed. While the unadjusted model (Model 1) yielded a positive association (HR = 1.49, 95% CI: 1.21 – 1.84), the adjusted model (Model 2) indicated a significant inverse association (HR = 0.76, 95% CI: 0.60 – 0.95, P = 0.0302). This reversal likely reflects confounding effects of baseline covariates such as inflammation, metabolic factors, or comorbidity burden, which were accounted for in the adjusted analysis.

**Figure 5** presents stratified trajectories by CKM subtype. Among patients whose first CKM event was T2DM, the subsequent risk of developing double CKM was significantly elevated (HR: 1.69, 95% CI: 1.21 – 2.36, P = 0.0022). The risk was even higher for those whose first event was CVD (HR: 2.31, 95% CI: 1.56 – 3.43, P < 0.001) or CKD (HR: 2.86, 95% CI: 1.85 – 4.41, P < 0.001). These subtype-specific transitions suggest that the type of initial CKM diagnosis modifies the subsequent risk of multimorbidity accumulation. However, across all subgroups, OA remained a consistent predictor of disease advancement.

Taken together, these findings demonstrate a clear and cumulative progression of CKM disease burden in patients with osteoarthritis, with dynamic effects observed from baseline through advanced multimorbidity stages. The visualization of transitions in **Figures 4**, **5**, alongside the full model specifications in **Table 3**, supports the conclusion that OA acts as a longitudinal clinical marker for systemic disease evolution.

### 3.4 Stacked transition probability analysis

**Figure 6:**
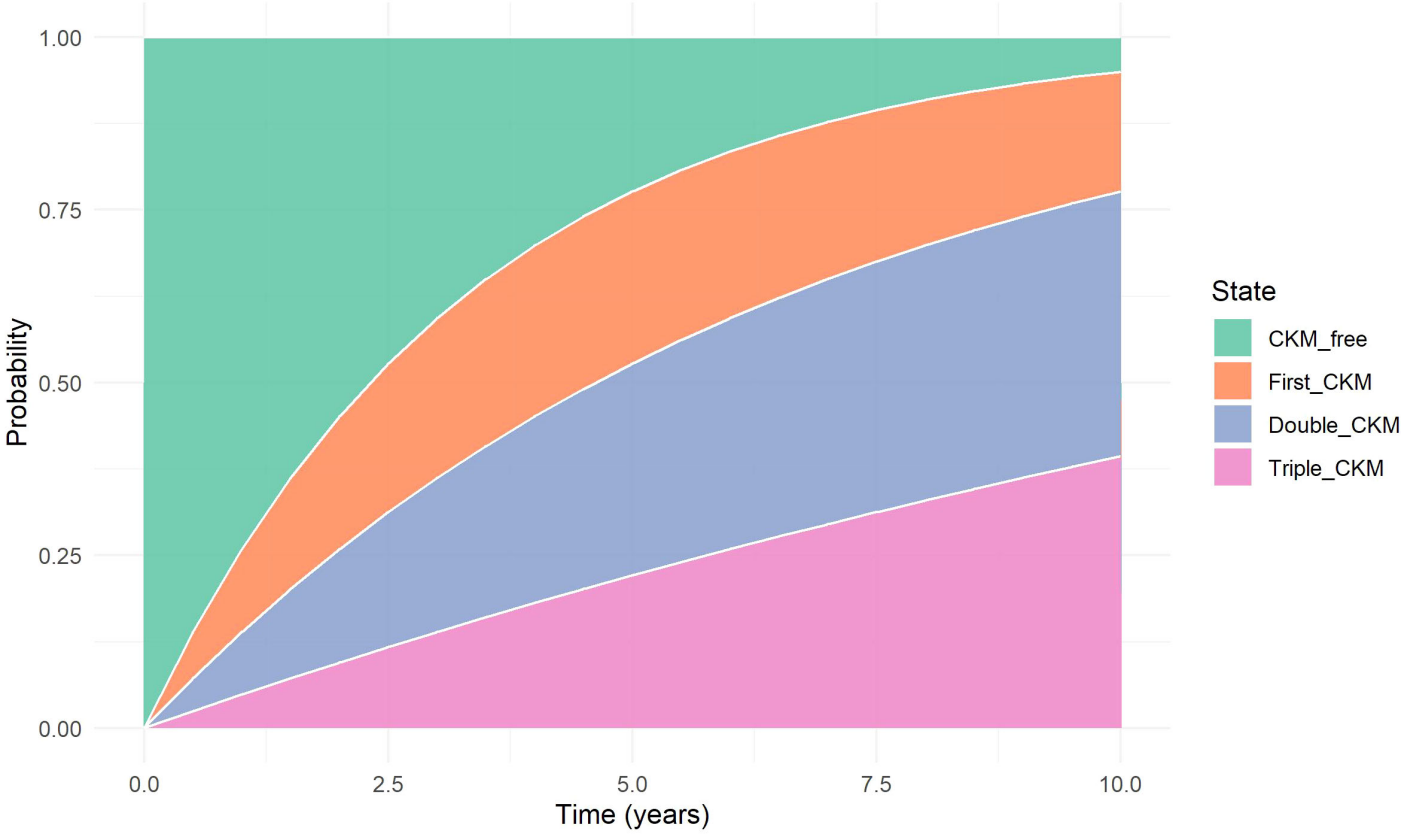
Stacked Transition Probability Plot. Stacked plot showing the cumulative probabilities of occupying each CKM disease state (CKM-free, first CKM, double CKM, and triple CKM) over time, as estimated from the progressive multi-state model. The visualization captures the gradual shift in state occupancy during follow-up, highlighting the accumulation of cardiometabolic conditions in a stepwise fashion.

To further illustrate the dynamic evolution of CKM multimorbidity, we estimated the cumulative transition probabilities for each disease state over time using a progressive multi-state model. The stacked transition probability plot (**Figure 6**) visually represents the distribution of the population across four disease states: CKM-free, first CKM, double CKM, and triple CKM.

At baseline, 100% of participants were CKM-free. Over time, the proportion of individuals in the CKM-free state steadily declined, accompanied by an increasing accumulation in the first, double, and triple CKM states. Notably, the growth of the triple CKM segment became more pronounced after approximately 5 years of follow-up, suggesting a nonlinear trajectory of disease clustering. This visualization reinforces the progressive nature of multimorbidity development and provides an intuitive understanding of transition dynamics at the population level.

### 3.5 Subgroup analyses by demographics and baseline CKM subtype

Subgroup analyses revealed that the association between osteoarthritis (OA) severity and CKM progression was consistent across age and sex strata but appeared more pronounced among participants with body mass index (BMI) ≥30 kg/m². This suggests that obesity may amplify the trajectory from OA to multimorbidity, possibly through shared inflammatory or metabolic pathways.

When stratified by baseline CKM subtype, notable differences in disease progression patterns were observed. Specifically, individuals with pre-existing cardiovascular disease (CVD) exhibited the highest likelihood of progressing to triple CKM multimorbidity, followed by those with chronic kidney disease (CKD), while participants starting with type 2 diabetes mellitus (T2DM) had a relatively lower proportion reaching triple disease status. These findings indicate distinct disease accumulation pathways depending on the initial metabolic condition.

**Figure 7:**
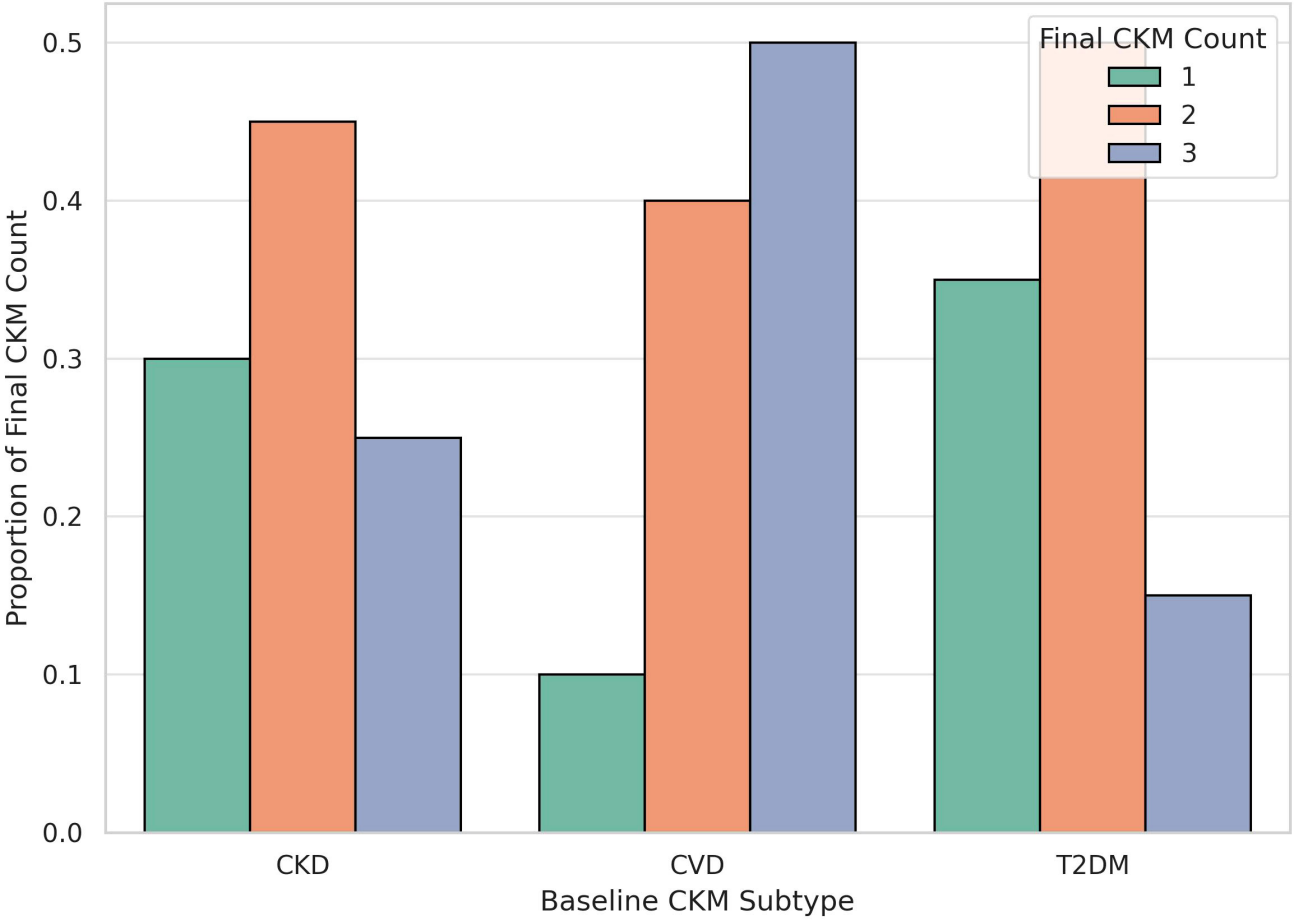
CKM Progression by Baseline CKM Subtype. The bar plot illustrates the distribution of final CKM disease burden (single, double, or triple multimorbidity) stratified by baseline CKM subtype (CVD, CKD, or T2DM). Patients with baseline cardiovascular disease (CVD) exhibited the highest proportion progressing to triple CKM, followed by those with chronic kidney disease (CKD) and type 2 diabetes mellitus (T2DM), suggesting heterogeneity in disease clustering pathways depending on initial disease type.

As shown in **Figure 7**, nearly half of those with baseline CVD progressed to triple multimorbidity, whereas CKD patients were more likely to transition to double CKM status. In contrast, most individuals with baseline T2DM developed either single or double CKM conditions, with a smaller proportion progressing to triple CKM. This heterogeneity in progression patterns highlights the importance of considering baseline disease phenotype in early risk stratification and personalized multimorbidity prevention strategies.

## 4 Discussion

In this population-based cohort study of adults with osteoarthritis (OA), we observed a strong association between OA severity and the development and progression of cardiovascular-kidney-metabolic (CKM) multimorbidity. A higher OA burden significantly increased the risk of incident CKM conditions and was independently linked to the progression from single to dual and eventually triple multimorbidity. Importantly, these associations remained robust after adjusting for demographic, behavioral, inflammatory, and metabolic factors. Our findings suggest that OA, traditionally viewed as a localized musculoskeletal disorder, may also serve as an early clinical indicator of systemic cardiometabolic dysfunction.

Given the retrospective and observational nature of this study, no causal inference can be drawn regarding the relationship between osteoarthritis (OA) and CKM multimorbidity. While the findings suggest a strong association, we did not employ advanced causal inference methods such as instrumental variable analysis, time-lagged exposure modeling, or propensity score adjustment. Future studies incorporating these methodologies are warranted to confirm the directionality and potential mechanistic pathways underlying this association.

Mechanistically, OA may contribute to CKM multimorbidity through several converging biological pathways. Chronic low-grade systemic inflammation, a hallmark of OA, has been implicated in endothelial dysfunction, insulin resistance, and renal microvascular injury—providing a shared substrate for cardiovascular, kidney, and metabolic diseases (14). Limited mobility due to joint pain and stiffness may further exacerbate obesity and metabolic syndrome, amplifying the risk of CKM clustering (15). In addition, metabolic abnormalities such as hyperglycemia and dyslipidemia have been shown to accelerate cartilage degeneration, indicating a bidirectional relationship (16). However, the lack of significant associations between OA and individual CKM components suggests that OA may exert its effects primarily through cumulative or synergistic pathways rather than isolated disease mechanisms.

Furthermore, this study employed a multi-state modeling approach to extend previous work by elucidating the temporal accumulation of CKM diseases among OA patients. We demonstrated that OA is not only associated with the initial onset of CKM conditions but also contributes to the clustering of multiple diseases over time. The observed risk gradient related to OA severity and BMI (17), especially the marked increase in risk during progression from single to multiple conditions, supports earlier findings linking elevated TyG index (18–20), metabolic syndrome, and OA with adverse cardiometabolic outcomes (21–24).

The selection of a progressive multi-state model over a conventional longitudinal model such as the linear mixed-effects model was driven by the discrete and sequential nature of the disease states under investigation. Our primary objective was to model the transitions between clinically defined cardiometabolic states—namely, from CKM-free to first CKM, then to double and triple CKM. These transitions occur at distinct time points and reflect categorical shifts in health status, which are not adequately captured by models designed for continuous outcomes. The multi-state framework enables explicit estimation of transition-specific hazards and accommodates the temporal ordering of disease accumulation, making it a more appropriate analytic approach for modeling multimorbidity trajectories in this context.

Clinically, our results support incorporating OA into early cardiometabolic risk stratification tools. Current CKM prevention frameworks focus mainly on traditional metabolic risk factors, but integrating musculoskeletal indicators such as OA—particularly among individuals with elevated BMI, hypertension, or reduced physical activity—may enhance risk prediction and promote earlier intervention. Moreover, lifestyle modifications like exercise and weight loss, which benefit both joint health and metabolic function, could provide dual advantages for OA patients at risk of multimorbidity (25).

This study offers several notable strengths. It draws on a well-characterized clinical cohort with standardized outcome definitions and employs robust statistical analyses using a multi-state modeling framework. All transition-specific Cox models within this framework converged without warnings or boundary issues, and the estimated coefficients remained stable across transitions, with no evidence of multicollinearity or inflated standard errors. The computation for each transition was efficient—typically requiring less than two minutes on a standard workstation—underscoring both the feasibility and the methodological rigor of the approach.

However, several limitations should be acknowledged. First, the observational nature of the study precludes definitive causal inference. Second, reliance on clinical records for disease ascertainment may introduce misclassification bias. Third, the absence of radiographic data, such as Kellgren–Lawrence grading, limited the ability to apply standardized, imaging-based definitions of OA severity. Fourth, residual confounding from unmeasured variables—such as the use of medications (e.g., NSAIDs, antihypertensives), dietary patterns, or socioeconomic status—cannot be excluded. Finally, because the cohort was drawn from a single geographic region in China, the generalizability of the findings to other populations may be limited. Future research should aim to replicate these results in more diverse cohorts and assess whether targeted interventions in patients with OA can alter the trajectory of CKM disease progression.

## 5 Conclusion

This study demonstrates that osteoarthritis severity is independently associated with increased risk of developing and progressing through CKM multimorbidity stages. Patients with higher OA burden were more likely to transition from no CKM condition to single, double, and triple disease states.

## Data Availability

The original contributions presented in the study are included in the article/**Supplementary Material**. Further inquiries can be directed to the corresponding author.
