# Supplementary material for: Osteoarthritis as a clinical marker of cardiovascular-kidney-metabolic multimorbidity: a population-based cohort study in China
Source: Front Endocrinol (Lausanne). 2025 Sep 9;16:1660319. doi: 10.3389/fendo.2025.1660319 (PMC12454107; doi:10.3389/fendo.2025.1660319)
Supplement: Supplementary file 1 [file DataSheet1.docx]

## Supplementary Materials

## Sensitivity Analysis: OA and Triple CKM

We conducted a single sensitivity analysis to evaluate the robustness of the observed inverse association between osteoarthritis (OA) and triple cardiovascular–kidney–metabolic multimorbidity (CKM) to potential bias from the competing risk of death. Because death status was unavailable, we constructed a surrogate mortality score based on age, sex, systolic blood pressure, C-reactive protein, and ESR. We then downweighted individuals with higher putative mortality probability (weights = 1 − predicted mortality, truncated at 0.05–0.95) in the adjusted logistic regression model. This approach approximates the scenario where high-mortality individuals may die before CKM progression, thus reducing the observed transition probabilities.

## Results

In the primary adjusted logistic model, OA was inversely associated with triple CKM, with an odds ratio (OR) of 0.628 (95% CI 0.522–0.755, p<0.001). In the sensitivity analysis that downweighted individuals with higher putative mortality risk, the association attenuated slightly to an OR of 0.654 (95% CI 0.543–0.788, p<0.001). This suggests that accounting for the competing risk of death would modestly weaken but not eliminate the observed inverse association. The E-value for the point estimate was 2.20 (E-value for the confidence limit closest to the null: 1.87), indicating that an unmeasured confounder associated with both OA and triple CKM by a risk ratio of at least these magnitudes would be needed to fully explain away the observed association.

## Supplementary Table S1. Sensitivity analysis of the association between osteoarthritis and triple CKM

| Model | OA OR | 95% CI low | 95% CI high | p-value | E-value |
| --- | --- | --- | --- | --- | --- |
| Primary (adjusted logistic) | 0.628 | 0.522 | 0.755 | <0.001 | 2.20 (CI: 1.87) |
| Sensitivity (downweighted high mortality) | 0.654 | 0.543 | 0.788 | <0.001 | — |

Abbreviations: OA, osteoarthritis; CKM, cardiovascular–kidney–metabolic multimorbidity; OR, odds ratio; CI, confidence interval. E-value represents the minimum strength of association, on the risk ratio scale, that an unmeasured confounder would need to have with both the exposure and the outcome, conditional on the measured covariates, to fully explain away the observed association. The sensitivity model downweights individuals with higher predicted mortality probability to approximate the effect of death as a competing outcome.
