# Supplementary figures and images for: Osteoarthritis as a clinical marker of cardiovascular-kidney-metabolic multimorbidity: a population-based cohort study in China
Source: Front Endocrinol (Lausanne). 2025 Sep 9;16:1660319. doi: 10.3389/fendo.2025.1660319 (PMC12454107; doi:10.3389/fendo.2025.1660319)

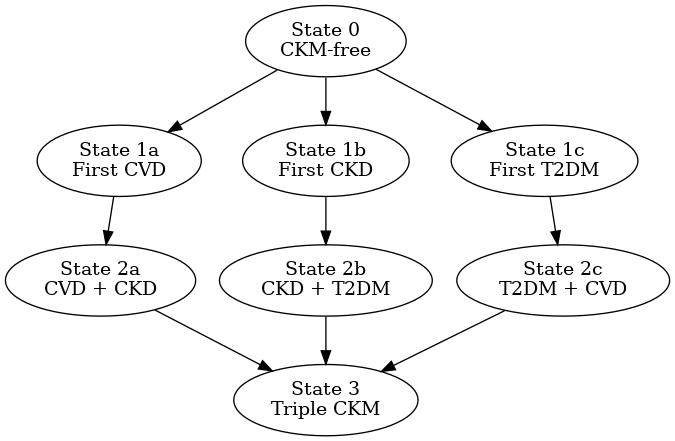

Supplement: Supplementary file 2 [file Image1.jpeg]
